# Optofluidic Waveguides for the Label-Free Study of Silk Protein Aggregates

**DOI:** 10.1021/acsomega.5c07826

**Published:** 2025-09-23

**Authors:** Jan R. Heck, Zenon Toprakcioglu, Tobias E. Naegele, Michael H. Frosz, Tuomas P. J. Knowles, Tijmen G. Euser

**Affiliations:** 1 Department of Physics, Cavendish Laboratory, 151956 University of Cambridge, CB3 0HE Cambridge, United Kingdom; 2 Yusuf Hamied Department of Chemistry, University of Cambridge, CB2 1EW Cambridge, United Kingdom; 3 Department of Engineering, University of Cambridge, CB3 0FA Cambridge, United Kingdom; 4 130458 Max Planck Institute for the Science of Light, Erlangen 91058, Germany

## Abstract

Methods for studying protein aggregation are crucial to understanding the associated disease pathologies and for functional biomaterial synthesis in nature and in the laboratory. The ideal measurement platform is low-volume, label-free, and noncontact, as well as easily integrated into continuous-flow microfluidic experiments to provide scalability. Current approaches realize only a subset of these requirements. Here, we demonstrate a new technique for studying protein aggregates and *in situ* aggregation within hollow-core photonic crystal fibers. These optofluidic waveguides allow us to perform continuous-flow microfluidic label-free analysis of silk fibroin protein in the form of preformed nanofibrillar aggregates and on the native protein as it undergoes aggregation *in situ* in the optofluidic waveguide. We demonstrate label-free ultraviolet absorbance measurements on both calibration-standard nanospheres and silk fibroin aggregates as well as monitoring the aggregation of native silk fibroin protein solution via simultaneous ultraviolet absorbance and intrinsic fluorescence measurements *in situ*. This technique forms a platform for the study of protein aggregation that is low volume, label-free, and optical, thereby providing a valuable optofluidic tool for a range of protein biophysics.

## Graphical abstract

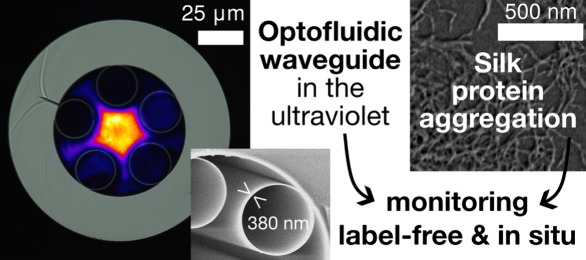

## Introduction

The aggregation of proteins, which involves the transition of soluble monomeric proteins into large insoluble structures that can reach up to micron sizes, has been associated with both biological function and malfunction.^1–3^ As such, it is of interest to a broad spectrum of research including utilizing proteins as building blocks for new materials and studying how disease-related proteins assemble and form amyloid structures. Examples of function include the self-assembly of silk from nanoscale monomer precursors, by which spiders and other arthropods create sophisticated functional biomaterials with impressive strength-to-weight ratios,^4^ while malfunction is exemplified by protein aggregation underlying the disease mechanism of several neurodegenerative pathologies, such as Alzheimer’s disease.^5^

### Silk Protein Aggregation Biophysics and Biomaterial Science

Silk is an excellent candidate for the study of protein aggregation, as it can be used to investigate the biophysical aggregation mechanisms starting from liquid silk protein solution^6^ while also being a functional biomaterial of interest due to its (bio)­engineering properties when aggregated into silk threads.^4,7,8^

Due to the laborious and resource-intensive procurement from natural precursors (typically reconstituted and purified from cocoons of the silkworm *Bombyx mori* in a multiday process^7^), the available protein sample volumes are limited. Yet current methods often rely on large-volume measurements, such as cuvettes (>1 mL) or well plates (>100 μL). In addition to resource requirements and sustainability concerns, these methods also present engineering challenges if they are to be used for studying samples under flow. However, continuous flow studies are essential for at least two reasons. First, they enable high-throughput studies that perform the large numbers of observations needed to explore the parameter space of experimental conditions. Second, flow-based experiments can induce and control shear forces, which are known to play a key role in aggregation processes.^9–11^ With silk, nature has optimized the process, resulting in impressive demonstrations of the ability to use flow-based shear forces in biomaterial synthesis. Besides silk worms, shear forces are also essential to the mechanism by which spiders exert intricate biophysical control of the aggregation of the protein solution flowing through their silk glands. This enables the on-demand extrusion of a solid web from a liquid silk solution in a highly efficient process. Hence, continuous-flow microfluidic approaches are essential tools for protein aggregation studies.

### Label-Free Aggregation Dynamics Monitoring

Noncontact optical methods are the preferred tool to monitor protein aggregation as it occurs (*in situ* and in real time).^6,12,13^ Unlike rheological measurements,^14,15^ they leave the proteins and their aggregation kinetics virtually unaltered. However, given the weak optical signature of nanometer-scale proteins, extrinsic fluorescent labels are typically introduced to increase the signal-to-noise ratio and simplify their detection, while some fluorophores also report their aggregation state.^2,16,17^ These fluorescent labels, such as the aggregation probe thioflavin-T (ThT) that binds to β-sheet structures, have recently been shown to influence protein–protein interactions and aggregation kinetics.^18,19^ Labeling also adds additional processing steps and inherently contaminates and perturbs the sample, thereby complicating downstream use or purification of the sample for synthesis applications.

These issues can be avoided by using label-free methods based on naturally occurring features of the sample, such as intrinsic fluorophores^20^ or their absorbance.^21^ However, without the benefit of purpose-engineered labels, label-free methods are generally less sensitive. At low concentrations, the reduced sensitivity must be compensated for by increased optical pathlengths, which then require higher sample volumes.

### Optofluidic Waveguides for Label-Free Optical Analysis

The optofluidic waveguides used in this work, known as hollow-core fibers (HCF) or antiresonant hollow-core fibers (AR-HCF), are a technology that enables long-pathlength (here, 17 cm) measurements using sample volumes that are in the microfluidic submicroliter regime.^22–24^ Their ability to guide light over long distances at the center of a microfluidic channel makes them ideal tools to perform label-free measurements at low protein concentrations (Figure 1).

**Figure 1:**
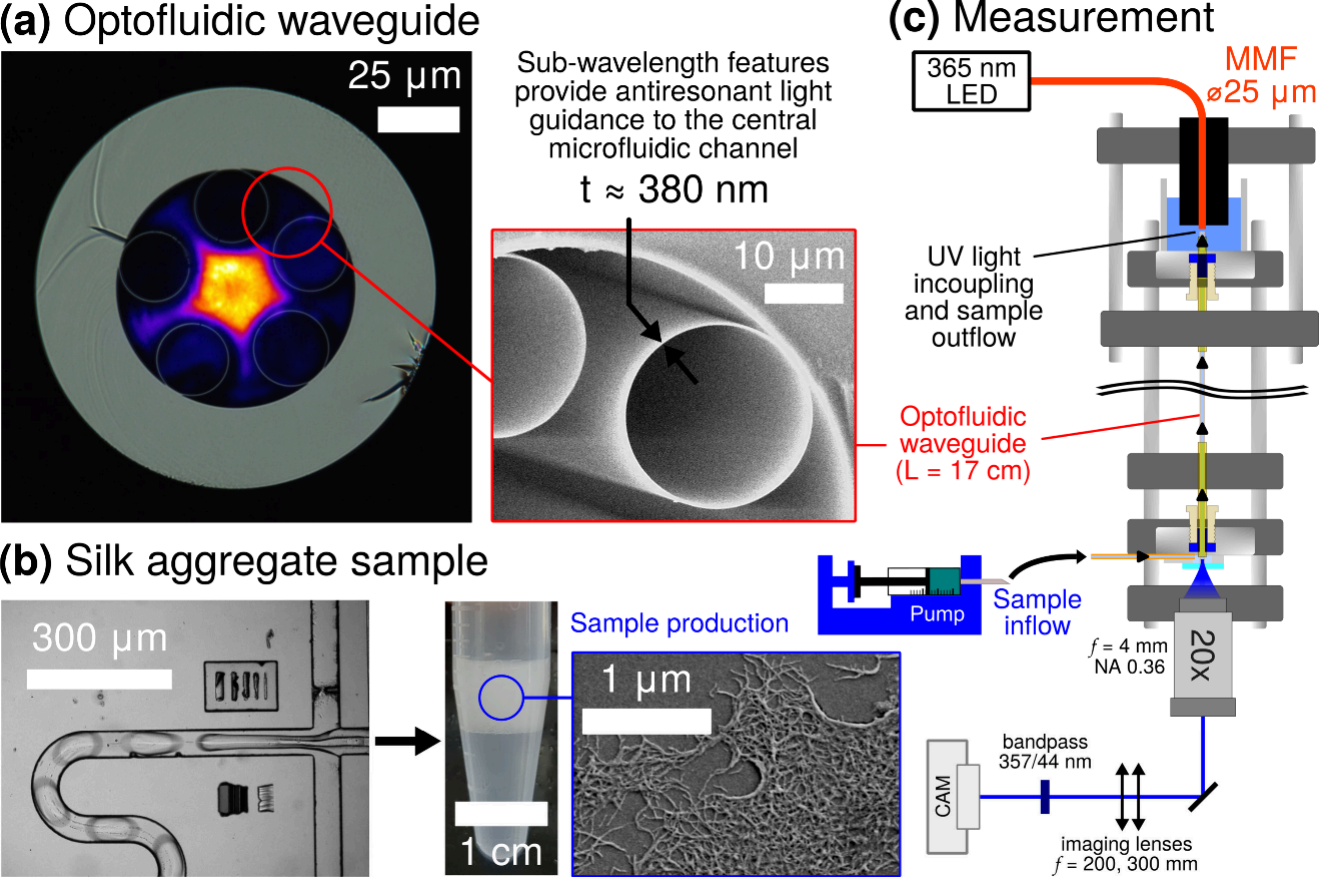
Principles of the optofluidic measurement platform. (a) Optofluidic HCF comprising a central microfluidic channel (*d* = 30 μm) surrounded by thin-walled capillaries that antiresonantly guide light along the channel. Core mode was observed at 350 nm wavelength after propagation through 17 cm of liquid-filled HCF. (b) Aggregates of silk fibroin protein are synthesized by droplet microfluidic coencapsulation of protein with ethanol acting as the aggregation inducer. (c) The resulting aggregate solution is analyzed with the optofluidic waveguide setup by measuring the ultraviolet extinction of the continuously flowing solution over a long pathlength (17 cm).

In biophysics, HCFs have been used for biomechanical studies through optofluidic trapping of red blood cells,^25^ the extended observation of phage motion enabled by long pathlengths,^26^ and for identifying cell viability via surface-enhanced Raman spectroscopy (SERS).^27^ For smaller objects on the molecular scale, biophysical measurements performed in HCF include the detection of the serum protein bovine serum albumin via either its intrinsic fluorescence^28^ or through its effect on waveguide transmission.^29^ In general, a range of spectroscopic methods have been adapted within HCF for biochemistry applications,^22^ such as detecting antibiotics via absorbance^30^ or SERS.^31^

### This Work

In this work, we use optofluidic HCF waveguides to study monomeric silk and its subsequent aggregation using two different methods. First, silk protein fibrillar aggregates formed *ex situ* (see Methods) are analyzed label-free via their optical (ultraviolet) absorbance, within the optofluidic waveguide, and under continuous flow. Second, we monitor native silk protein aggregation *in situ* the waveguide by simultaneously tracking the absorbance as before while additionally measuring the protein intrinsic fluorescence from tryptophan residues.^28,32^

## Results and Discussion

The method’s reproducibility was tested by measuring the absorbance of calibration standard polystyrene spheres (*d* = 41 ± 4 nm, NIST 3040A) under continuous flow (1050 μL/h). First, an aqueous solution was prepared from the stock and measured in a large-volume UV–vis instrument at 365 nm (1.5 mL cuvettes, Shimadzu UV-3600i Plus). This solution was then diluted to OD 0.004/cm (corresponding to *T* = 86% over 17 cm, the optical pathlength of the optofluidic waveguide) to serve as the test sample. The resulting measurement series, repeatedly alternating the flow through the optofluidic waveguide between sample and water, resulted in *T* = (88.2 ± 0.4)% across the four measurements (Figure 2).

**Figure 2:**
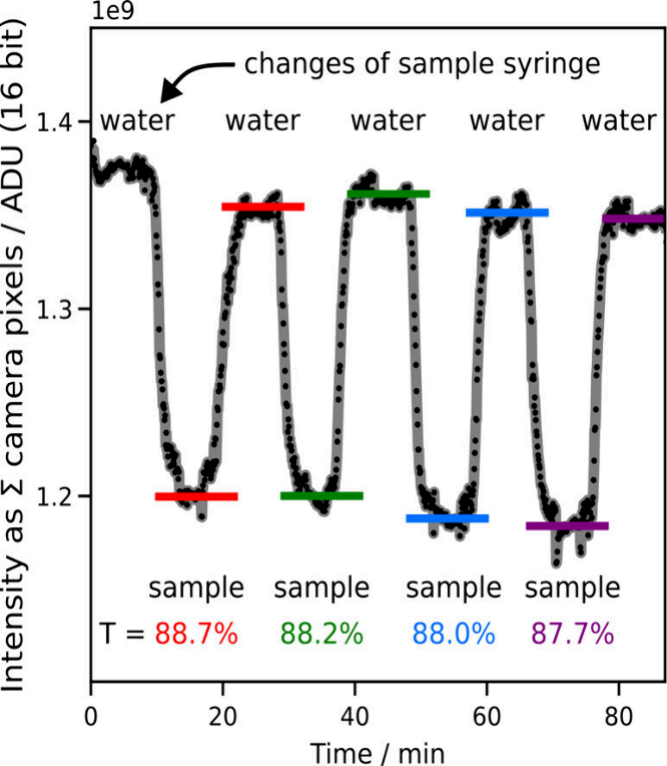
Reproducibility of the ultraviolet absorbance measurement of the optofluidic waveguide under continuous flow. The sample, polystyrene nanospheres (*d* = 41 ± 4 nm), is alternated with water by exchanging the syringe feeding the microfluidic circuit. The transmitted intensity is decreased by the less transparent sample solution, quantified by the ratio of transmitted intensity (colored bars) through a given sample relative to water.

### Silk Fibril *ex Situ* Aggregates Measured via Absorbance

The absorbance of four different concentrations of the *ex situ* aggregated silk fibroin solution was measured in triplicate under continuous flow according to the protocol described in the Methods. The stock solution from the *ex situ* aggregation process was diluted twice to yield a sufficient volume for a large-volume (1.5 mL cuvette) UV–vis reference measurement. This sample was then used to create a series of dilutions (1:1 with water each time). For each dilution, a glass syringe containing the sample was fed through the optofluidic waveguide system at 300 μL/h, followed by a syringe containing water at the same flow rate. This swapping procedure was performed three times to yield a triplicate. The transmitted light intensity was recorded after each syringe change once the reading had stabilized, typically 15 min. As the exact concentration *C*_0_ of the stock solution after filtering was unknown (the concentration of the initial silk fibroin solution before filtering was 1 g/L; see Methods), the absorption data in Figure 3 are plotted against relative concentration C/C_0._

**Figure 3:**
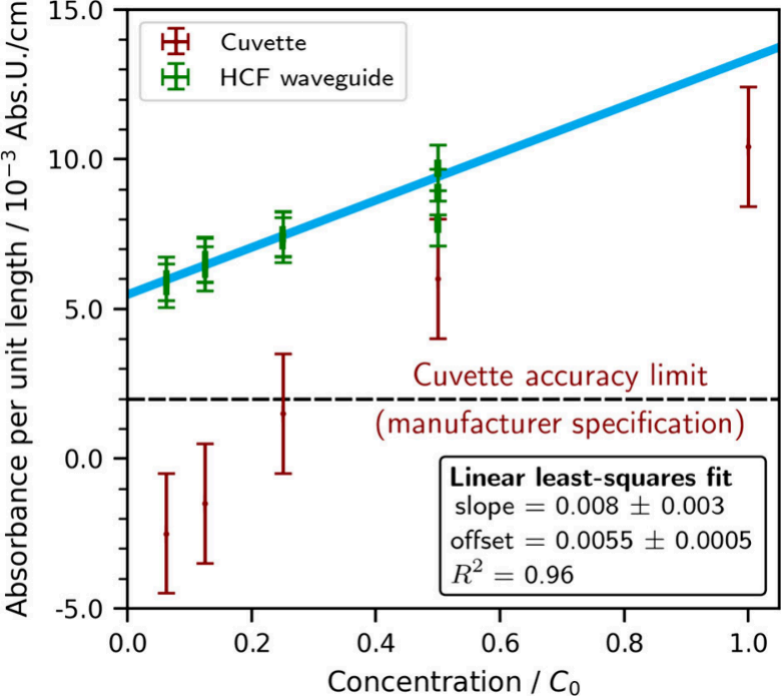
Ultraviolet extinction measurements on silk aggregates. The optofluidic waveguide (HCF) setup was used to measure a dilution series in triplicate for each aliquot (green), which was also measured in large-volume (1 cm) cuvettes (red). Note that lower concentrations in the cuvette UV–vis spectrophotometer fell below the manufacturer’s detection limit.

The results (Figure 3) show good linearity (*R* ^2^ = 0.96) for the HCF measurements across the entire concentration range. Using the standard definition for the LoD as 3.3 σ/(slope of the linear fit), where σ is the averaged standard error of all waveguide absorbance measurements, a value of 0.12 *C*/*C*_0_ was obtained. This relative concentration corresponds to an absolute silk fibroin concentration of, at most, 0.12 g/L (using an upper limit for *C*_0_ of 1 g/L).

By comparison, the cuvette reference measurements for concentrations *C*/*C*_0_ < 0.25 result in absorbance values below the detection limit of the cuvette spectrometer (OD 0.002/cm), despite requiring a thousand times higher sample volume.

The highest concentration (1 *C*_0_) measured in cuvette was also attempted in HCF, despite the 17 times longer pathlength leading to high OD (nearly complete absorption). The spread of absorbance values increased significantly, reaching beyond two standard deviations. Additionally, at high concentrations, silk aggregates become permanently adsorbed onto the fiber, reducing the transmission and preventing the light transmission from fully recovering upon water flushing, contributing an offset accumulating over time. This adsorption of protein onto HCF is known and has been characterized in the literature.^29^ Hence, this sample was considered to be too concentrated to be measured by the HCF and was excluded.

### Silk Aggregation Characterization by Absorbance and Fluorescence Spectroscopy

Next, we studied the aggregation of the native liquid silk solution within a cuvette. Silk fibroin solutions derived from natural-origin precursors exhibit considerable batch-to-batch variation of aggregation behavior (initial concentration and distribution of oligomeric seeds).^9,14^ To characterize our batch, we performed large-volume absorbance and fluorescence measurements over a period of several days (Figure 4). With progressing aggregation, the initially fully transparent silk was observed to take on a cloudy-white color and soft gel consistency (Figure S6).

**Figure 4:**
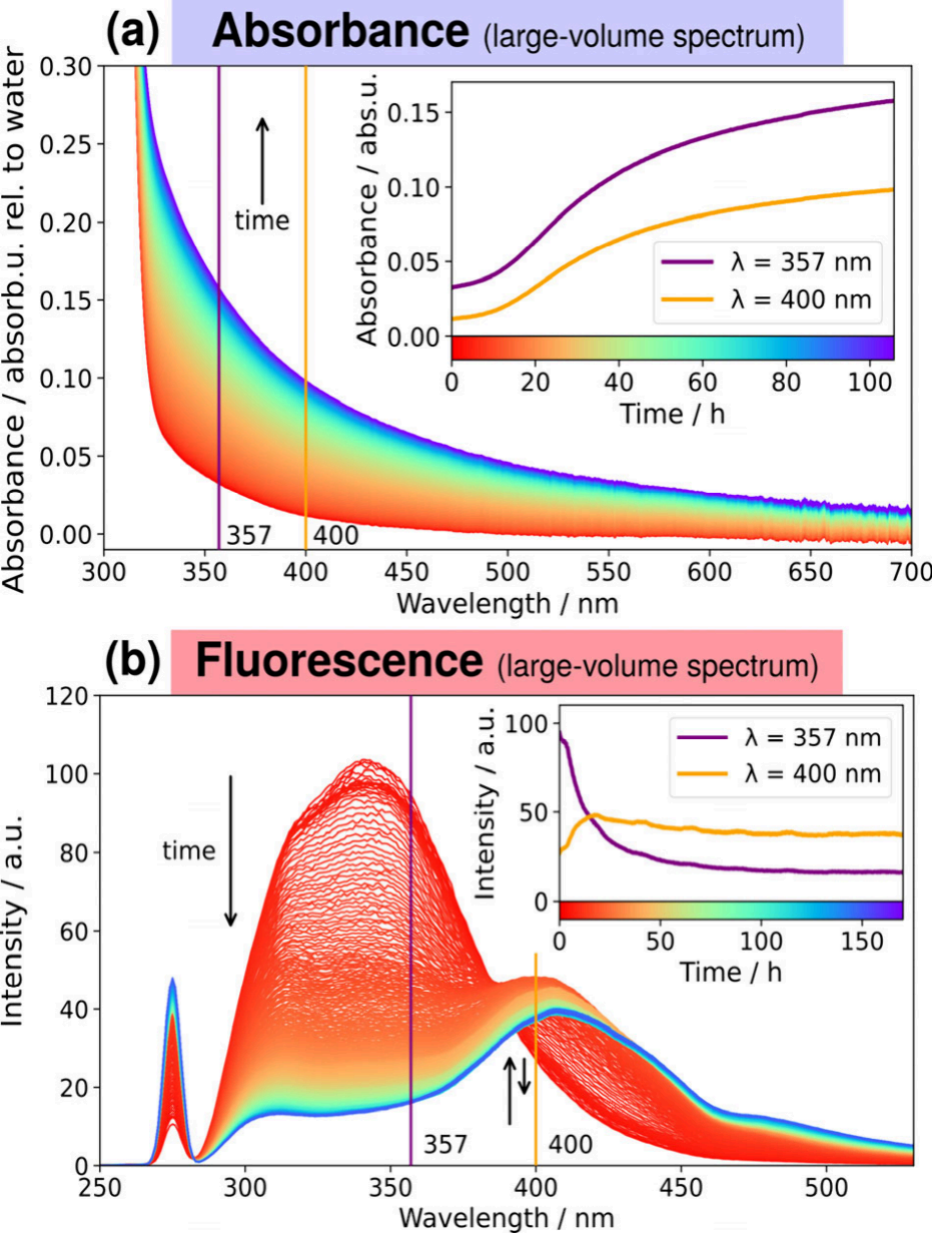
Silk protein aggregation dynamics in a cuvette. Aggregation is induced for silk fibroin solutions with ethanol (see text). The resulting aggregation dynamics are monitored in cuvette over several days via (a) absorbance and (b) intrinsic fluorescence excited at 275 nm. Insets show the timecourse of the indicated wavelengths, 357 and 400 nm.

The UV–vis measurements on the aggregating sample (Figure 4a) show a monotonic increase in absorbance over the entire period across all wavelengths above 310 nm. To quantify this increase, two wavelengths were tracked over time: 357 nm (the center of the bandpass filter used in the optofluidic waveguide experiments), and 400 nm (an emerging visible fluorescence peak, discussed below). At wavelengths below 310 nm, the absorbance was shown to slightly decrease (Figure S8).

Both show a sigmoidal shape characteristic of protein aggregation,^2^ with an inflection point at ca. 24 h. Absorbance (which is understood as a combined contribution from scattering and molecular absorption) is higher at shorter wavelengths, with the absorbance measurement at 357 nm being around twice as high (in logarithmic units of optical density) as that for 400 nm. This means that the bandpass-filtered (357 ± 44) nm wavelength band being tracked via absorbance is a monotonically increasing indicator of aggregation.

The fluorimetry spectra on the aggregating sample (Figure 4 b) can best be described as the superposition of two overlapping spectral features evolving over time, corresponding to silk fibroin monomer and aggregated species, as reported in the literature.^33^ The broad fluorescence emission peaked at ca. 345 nm is identified as the silk’s intrinsic fluorescence, originating primarily from the tryptophan residues in the silk fibroin protein, and dominates the spectrum initially. As aggregation progresses, a visible fluorescence emerges due to molecular cross-linking,^33^ showing first as a shoulder on the UV tryptophan peak and eventually becoming a separate peak centered on ca. 400 nm. When tracked over time, the visible fluorescence peak dominates over the UV peak from ca. 15 h onward, after which it decreases by ca. 20% and plateaus. Finally, the narrow peak at 275 nm corresponds to scattered excitation light, which is observed to increase during the aggregation process.

Even though the tryptophan (UV) fluorescence is expected to increase with aggregation state,^33^ it is seen to decrease in our data. This may be due to the significantly different aggregation conditions in the *ex situ* studies of ref 33, who used a much higher silk concentration of 80 g/L (compared to 1 g/L here) and used 3D scaffolds to prevent formed aggregates from sedimenting at the bottom of the sample cell. In our experiment, formed aggregates are more likely to sediment at the bottom of the cuvette away from the irradiated detection region, thus reducing the observed fluorescence intensity. Stirring could alleviate this effect but would significantly affect and modify the aggregation process, as well as being difficult to implement for μL volumes (giving another motivation for continuous-flow microreactors as presented here).

Comparing the interval *t* = 5 to 20 h (corresponding approximately to the onset and inflection point of the aggregation), the fluorescence at 357 nm decreases by 50%, whereas the absorbance at the same wavelength increases by 79%. Over the same period, the 275 nm peak in Figure 4b more than doubles, indicating an increased scattering of the pump light. These observations are consistent with our assumption that the aggregates increase in size, with part of them sedimenting at the bottom of the cuvette. The fluorescence intensity is proportional to the (decreasing) amount of silk in the irradiated part of the setup. The scattering intensity (and therefore the observed absorption/extinction), on the other hand, can still increase. This is because the Rayleigh scattering cross-section of a (silk) particle with radius *r* is expected to scale as *r* ^6^, while its volume only scales with *r* ^3^. A smaller mass of silk aggregates can, thus, result in an increased extinction. Hence, not all of the fluorescence decrease is accounted for purely by increased self-absorption, i.e., the fluorescence decreased even when accounting for the self-absorption effect.

### Silk Aggregation in the Optofluidic Waveguide Monitored via Absorbance and Intrinsic Fluorescence

Figure 5 shows the optofluidic waveguide aggregation experiment combining transmission and fluorescence measurements by alternating between their different excitations. Hence, both measurements are incorporated *in situ* by toggling between the two modes of excitation and taking a camera image for each (Figure 5a, described in more detail in Methods).

**Figure 5:**
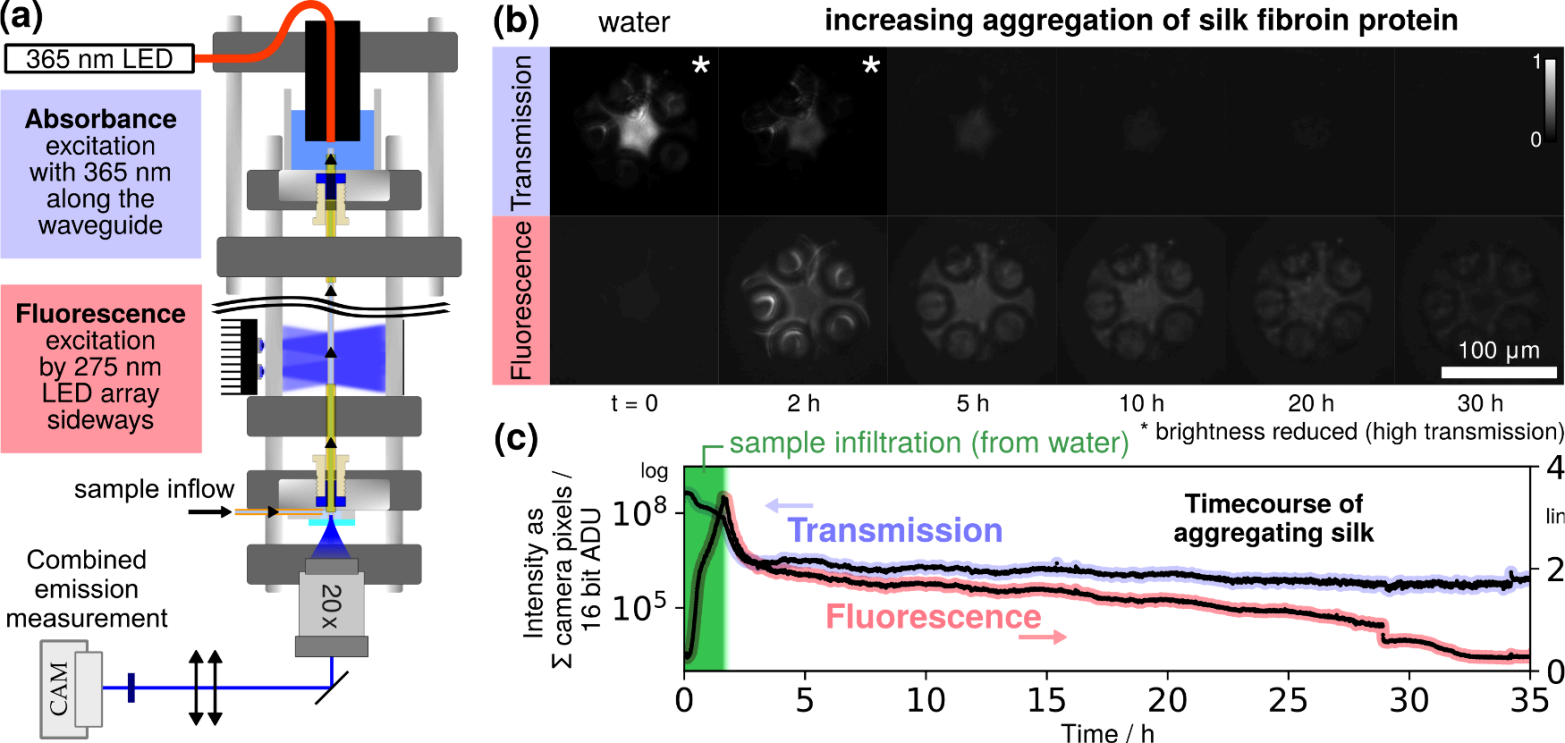
Silk aggregation was monitored *in situ* in the optofluidic waveguide via UV absorbance and intrinsic fluorescence. (a) Principle of combined measurement of absorbance (recorded as transmitted light) and intrinsic fluorescence by alternating light sources with a shared emission measurement (see the text). A 357/44 nm bandpass filter was placed in front of the camera. The aggregation of silk under continuous flow is then (b) monitored by imaging, from which an (c) integrated intensity timecourse is calculated. When initially (*t* = 0) filled with water, the optofluidic waveguide channel exhibits only weak background fluorescence and guides UV light with little absorbance. When the silk protein sample is introduced (*t* > 0), intrinsic protein fluorescence appears and absorbance increases. ADU: analog-to-digital unit (camera pixel values).

We define the start time of the experiment *t* = 0 as the moment at which the water-filled syringe was replaced with one containing silk. The silk solution itself was prepared 2 h beforehand, so the aggregation in fact begins from *t* = −2 h onward. For the timecourse, both measurements are background-subtracted by their respective minimal values. For display (but not the timecourse), the images are intensity-scaled as described in the to better visualize the large changes in intensity.

In agreement with the large-volume measurements, the UV absorption was observed to increase throughout the aggregation experiment (i.e., transmission decreases). The transmission eventually plateaus near the sensitivity limit (noise floor) of the camera. Besides a better detector, the Outlook section proposes techniques to significantly improve this limit by modulating the excitation power relative to the sample absorbance.

The UV (primarily tryptophan) fluorescence is seen to increase until ca. 2 h, after which it decreases for the remainder of the experiment. A near-instantaneous drop of ca. 10% observed toward the end of the experiment (*t* = 29 h) is identified as a formed aggregate coming loose and being flushed out of the fiber, thereby reducing the amount of fluorescence.

### Silk *ex Situ* Aggregates

Silk aggregates produced *ex situ* in a microfluidic chip were analyzed in the optofluidic waveguide by measuring their absorbance (reduction in transmission). This analysis showed good linearity (*R* ^2^ = 0.96) down to a 16-fold dilution. By comparison, despite the thousand-fold larger sample volume and lack of continuous flow operation, the same samples measured by a large-volume UV–vis fell below its LoD. The increase in sensitivity for the HCF measurement can be explained by the much larger optical pathlength in the optofluidic waveguide (17 cm) compared to the cuvette (1 cm), which by the Beer–Lambert law gives the weakly absorbing samples an exponential increase in signal (larger fraction of light absorbed).

### Silk Aggregation inside the Optofluidic Waveguide

As a proof of concept, the optofluidic waveguide was used to measure the label-free absorbance and intrinsic fluorescence of aggregating silk *in situ* and under flow. The system demonstrated that such a measurement is possible over the long time scale of protein aggregation that results from such low concentrations.

While the absorbance shows the expected trend of increased absorptivity over time, the intrinsic fluorescence behavior differed from that observed in the cuvette, being seen to first increase before it begins to decrease. The majority of this effect is accounted for by the time that it takes for the silk to displace the water in the optofluidic waveguide, during which the fluorescence increases because more silk is introduced. By comparison to the earlier *ex situ* aggregate data (Figure 2) performed at a 35 times higher flow rate, linear extrapolation would suggest an exchange time of 3 h, which is larger than observed. However, the brief interruption of flow after a syringe change and the subsequent time required to re-establish a pressure equilibrium by engaging the syringe plunger introduce an additional unknown factor, which at higher flow rates (faster syringe plunger speed) becomes less pronounced. Hence, both effects combined could explain the observed filling time. Automated and zero-dead-volume sample loading loops could alleviate these prototype issues.

Unlike in the ambient pressure cuvette experiments, the silk solution in the optofluidic waveguide is under pressure and is not in contact with air. Even small changes in shear force and pressure affect protein aggregation,^34–36^ as do interfaces between liquid and air, which have been shown to produce aggregates of silk when dispensed from syringes.^37^ Moreover, the air interface itself can act as a nucleation promoter and enhance protein aggregation, in general. Therefore, it is taken as support for the hypothesis that the aggregation of the silk in the microfluidic circuit of the optofluidic waveguide does not follow the same kinetics as the cuvette experiment. Further research is needed to study the effects of pressure and shear forces on aggregation, which dominate in this experimental system compared with more conventional cuvette-based measurements, making it at the same time suited for this research.

## Conclusion

The method developed and presented here, based on an optofluidic waveguide (a HCF) for measuring the ultraviolet absorbance and intrinsic fluorescence of proteins and their aggregation states, was employed for the study of silk in two examples: the quantification of *ex situ* formed silk fibroin aggregates via their ultraviolet absorbance, and the monitoring of an aggregating silk fibroin protein solution through both intrinsic fluorescence and ultraviolet absorbance *in situ*. In both cases, the method allowed measurements to take place label-free in a microfluidic system under continuous flow at a low concentration. This makes the presented work a new and valuable tool for the study of protein aggregation.

## Outlook

### Optofluidic Waveguides as a Platform for Protein Aggregation Biophysics

Protein aggregation remains an active area of research, where new methods can contribute by adding to the existing toolkit available to the biophysics researcher, thereby posing and answering new questions. The method presented here used a long pathlength optofluidic waveguide (a HCF) to allow for label-free yet sensitive optical measurements *in situ* a continuous flow microfluidic setup.

Continuous flow systems such as the one presented here can give real-time feedback on *in situ* aggregation experiments. A key advantage is that such systems inherently allow the impact of any experimental interventions on the protein aggregation pathway to be followed online, and actions taken accordingly. For example, the system could be adapted to increase or decrease the shear forces (by altering the flow rate) at different stages of aggregation, and observing the change in aggregation kinetics. This time-varying shear force is a process also seen in the production of spider silk, and this platform allows new functional biomaterial synthesis pathways modeled on that observed in nature to be studied.^6,38^

The method presented here is able to reduce the required sample volumes at a given concentration while maintaining sensitivity at levels of large-volume experiments, which can be used to push the limits of the lowest concentration samples that can be studied. This, in turn, may unlock new insights, especially for exploring the kinetics of low concentrations and the onset of aggregation. These earliest stages of aggregation are also the most important in determining protein aggregation dynamics, yet demand high sensitivity since the oligomeric protoaggregates are challenging to observe due to their small size.

### Flow Systems Give the Potential for Downstream Processing and Recirculation

An advantage of continuous flow experiments such as the one presented here is that their outflow can be processed or analyzed downstream while the experiment continues uninterrupted. Unlike in static systems, such as well plates, sample extraction is an inherent feature of continuous-flow systems and does not disturb the experiment. For example, the sample exiting the optofluidic waveguide could be collected for phenotype (morphology) analysis by SEM at different stages of aggregation. Correlating the morphology of the aggregates with the absorbance and fluorescence characteristics of the sample then allows for new insights from the resulting data and confident association of absorbance and fluorescence measurements with the underlying biophysical changes.

The experimental system presented here is especially suited to exploring the effects of shear forces on protein aggregation. The high aspect ratio in the optofluidic waveguide results in a uniform (due to high hydrodynamic compliance) environment, where applied pressures can cover a wide dynamic range. Therefore, the system’s continuous flow nature would allow time-varying shear forces to be applied by varying the flow rate (applied pressure) and observing the effects in real time. A comparison to microrheology methods can validate this method. For achieving even smaller sample volumes, the protein could be recirculated through the optofluidic waveguide, either in a closed-loop circuit or by back-and-forth flow with reservoirs on both sides. Finally, for proteins with challengingly slow aggregation kinetics (e.g., days or months at room temperature), time scales are typically accelerated by using above-physiological concentrations or induction via heat and chemicals (e.g., pH change). Focusing instead on higher shear forces while retaining online analysis of the aggregation state would open up low-concentration studies for these long-time scale aggregation processes, which unlike chemical induction methods can be rapidly paused at any stage of the aggregation.

### High Dynamic Range Absorbance Measurements and Spectral Analysis

In the experiments presented, absorbance measurements are currently limited by the low light transmission at the high absorbance encountered in the later stages of aggregation. A technical improvement in the form of closed-loop feedback control of the excitation power would lead to a significantly increased sensitivity. That is, instead of keeping the excitation light intensity fixed at the maximum level that does not oversaturate the camera at the beginning of the experiment, the LED’s power could be continuously increased to compensate for the higher absorbance. This would keep the transmitted light at a similar level and, therefore, be sensitively detectable, with data analysis referencing the transmitted light to the LED output power.

The system is not limited to bandpass-filtered fluorescence microscopy as presented here but can incorporate spectroscopy (or hyperspectral imaging), as we have shown before.^28,32^ The limiting element is typically the sensitivity of the available detector (spectrometer). Besides more sensitive detectors, based on using a waveguiding geometry, a path toward enabling this with the current system is for the fluorescent excitation volume to be increased further by using a longer length of HCF, trading sample volume for sensitivity.

If spectroscopic analysis is achieved by this increased sensitivity, then the multiple fluorescence peaks and the full absorbance spectrum can be tracked over time. Rich in structural information that can be understood via molecular dynamics models,^33^ this can contribute to a biophysical understanding of the underlying protein aggregation kinetics pathways.

## Methods

### Optofluidic Waveguide

The optofluidic waveguide used was a custom-designed antiresonant hollow-core fiber^39^ that guides ultraviolet light in water (transmission maximum ca. 350 nm). As shown in Figure 1a, the design surrounds a central microfluidic channel (*d* = 30 μm) with five silica glass capillaries (*d* = 25 μm), which have their wall thickness (*t* = 380 nm) matched to achieve antiresonant light guidance within the central channel according to the antiresonant interference condition of order *m*,

$$\lambda_{m}^{\mathrm{ar}}=\frac{4t}{2m+1}\sqrt{n_{\mathrm{silica}}^{2}-n_{\mathrm{sample}}^{2}}$$

where *n*_silica_ and *n*_sample_ are the (wavelength-dependent) refractive indices of the HCF’s glass and the infiltrated sample, respectively. The optical transmission properties of the HCF used in this work are discussed in detail in refs 28 and 32 (see also Figure S3).

### Microfluidic and Light Coupling

To operate the HCF as an optofluidic waveguide, it is mounted into a custom-built fixture that combines optical access with a microfluidic connection (Figure 1c). Inside the HCF, light and fluid traverse in opposite directions. On the top (the liquid outflow and light incoupling side), a large-volume (>2 mL) open reservoir is filled with water. A core size 25 μm multimode fiber (MMF, size matched to the HCF core) is used to butt-couple ultraviolet light from an LED into the optofluidic waveguide (separation between the fibers less than 20 μm) within this reservoir. After traversing the sample-filled optofluidic waveguide, the transmitted light emerges from the distal end (bottom side) within a low dead volume (<2 μL) chamber sealed by a quartz window. The end facet is observed through a custom-built inverted microscope, with all optics used being UV-grade. The power of the light impinging on the sample was kept below 0.5 μW and is characterized spectrally in Figures S3 and S4.

### Silk Aggregates Production *ex Situ*

Monomeric silk solution was purified from a natural source using an established protocol.^7^ In brief, silk cocoons are cut into pieces and degummed. The resulting fibers are dissolved using lithium bromide before performing dialysis to purify the silk fibroin. To form fibrillary protein aggregates *ex situ*, the silk fibroin solution was coencapsulated with ethanol into microfluidic droplets. The concentrations and flow rates used for the synthesis were silk fibroin solution (1 g/L) at 100 μL/h, ethanol (96%) at the same flow rate, and FC-40 oil with a surfactant at 200 μL/h. After postprocessing the output of the microfluidic chip by repeated displacement of the surfactant as per the protocol in ref 40, the now aqueous sample was passed through a membrane filter (0.45 μm), done slowly (>30 s) to minimize shear forces. This step will filter out some fraction of the solution’s protein content such that we refer to the resulting stock as having concentration *C*_0_, with *C*_0_ < 1 g/L. The resulting aggregate morphology was characterized by scanning electron microscopy (SEM) and was found to be fibrillar in nature, ranging from nanometers up to several microns in size (Figure 1b). Further SEM images are shown in Figure S5.

### Absorbance Measurement in the Optofluidic Waveguide

Absorbance measurements were taken using the following procedure, which found the ratio of transmission through a sample relative to water. The intensity of UV light emerging from the HCF is recorded by summing the pixel values of the camera image (Prime 95B, Teledyne Photometrics). With the sample to be measured currently flowing through the optofluidic waveguide, the syringe feeding the microfluidic circuit is swapped to one containing water, acting as the transmission reference. Then, the transmission ratio, defined as *T* = (light intensity with the sample present)/(light intensity after water replaced the sample), was calculated to yield a measure of absorbance for the sample.

### Silk Aggregation *in Situ* Protocol

To demonstrate the ability to monitor the aggregation of liquid silk fibroin solution *in situ*, the absorbance measurement described above was multiplexed with a label-free intrinsic fluorescence measurement similar to the one we have described previously.^28,32^

In brief (cf. Figure 5a), irradiation from the side over a length of ca. 5 cm with 275 nm UV light is used to excite the intrinsic fluorescence of the sample, originating primarily from the silk’s tryptophan residues.^41^ The emitted fluorescence is then collected and guided by the waveguide to be imaged and measured with the same shared optical detection setup as that for the absorbance measurements. For this to be possible, the wavelength for the absorbance measurements (λ = 365 nm) was intentionally chosen to be close to that of the intrinsic fluorescence emission of tryptophan (λ = 350 nm), such that both fall within the chosen bandpass filter (λ = (357 ± 44) nm). Measurements were alternated between absorbance (excitation guided along the waveguide) and fluorescence (excitation from the side), with images of 10 s exposure time recorded every 15 s (i.e., two data points of the same type are 30 s apart). To prevent fast aggregation, given that the shear rate is nonuniform throughout the channel (being highest at the surface of the channels), the flow rate was set to 30 μL/h, i.e., 10 times lower than in the previous experiment.^42^ Reference measurements of aggregation dynamics (Figure 4) were taken with a commercial right-angle cuvette fluorometer (Varian Cary Eclipse).

Aggregation was induced before the start of the experiment by diluting the aqueous silk fibroin stock with ethanol, similar to the *ex situ* aggregate formation described above, to a final concentration of 5 g/L silk and 40% ethanol. Mixing was performed by gentle pipetting to reduce shear forces and limit the progress of aggregation during this step.

## Supplementary Material







## Data Availability

The data that support the findings of this study are available from the corresponding author upon reasonable request.

## Notes

Photographs of the experimental setup, UV–vis characterization of the silk aggregates, transmission characterization of the optofluidic waveguide, spectrum of the light source, nanofibrillar silk aggregate morphology imaged with SEM, photographs of silk aggregated in cuvette, and information onf time-lapse videos of *in situ* transmission and fluorescence of aggregating silk (PDF)
Timelapse video of the in situ aggregation of silk in the HC-PCF, observed via ultraviolet transmission (MP4)
Timelapse video of the in situ aggregation of silk in the HC-PCF, observed via label-free ultraviolet fluorescence (MP4)
