# Supplementary material for: Optofluidic Waveguides for the Label-Free Study of Silk Protein Aggregates
Source: ACS Omega. 2025 Sep 23;10(39):46115–23. doi: 10.1021/acsomega.5c07826 (PMC12509126; doi:10.1021/acsomega.5c07826)
Supplement: Supplementary file 1 [file ao5c07826_si_001.pdf]

Optofluidic waveguides for the label-free study of silk protein aggregates

Jan R. Heck,[1,2] Zenon Toprakcioglu,[2] Tobias E. Naegele,[3] Michael H. Frosz,[4] Tuomas P. J. Knowles,[2] and Tijmen G. Euser[1*]

1 Department of Physics, Cavendish Laboratory, University of Cambridge, CB3 0HE, Cambridge, United Kingdom

2 Yusuf Hamied Department of Chemistry, University of Cambridge, CB2 1EW, Cambridge, United Kingdom

3 Department of Engineering, University of Cambridge, CB3 0FA, Cambridge, United Kingdom

4 Max Planck Institute for the Science of Light, Erlangen, Germany

* To whom correspondence should be addressed: te287@cam.ac.uk

Supporting information

Fig. S1

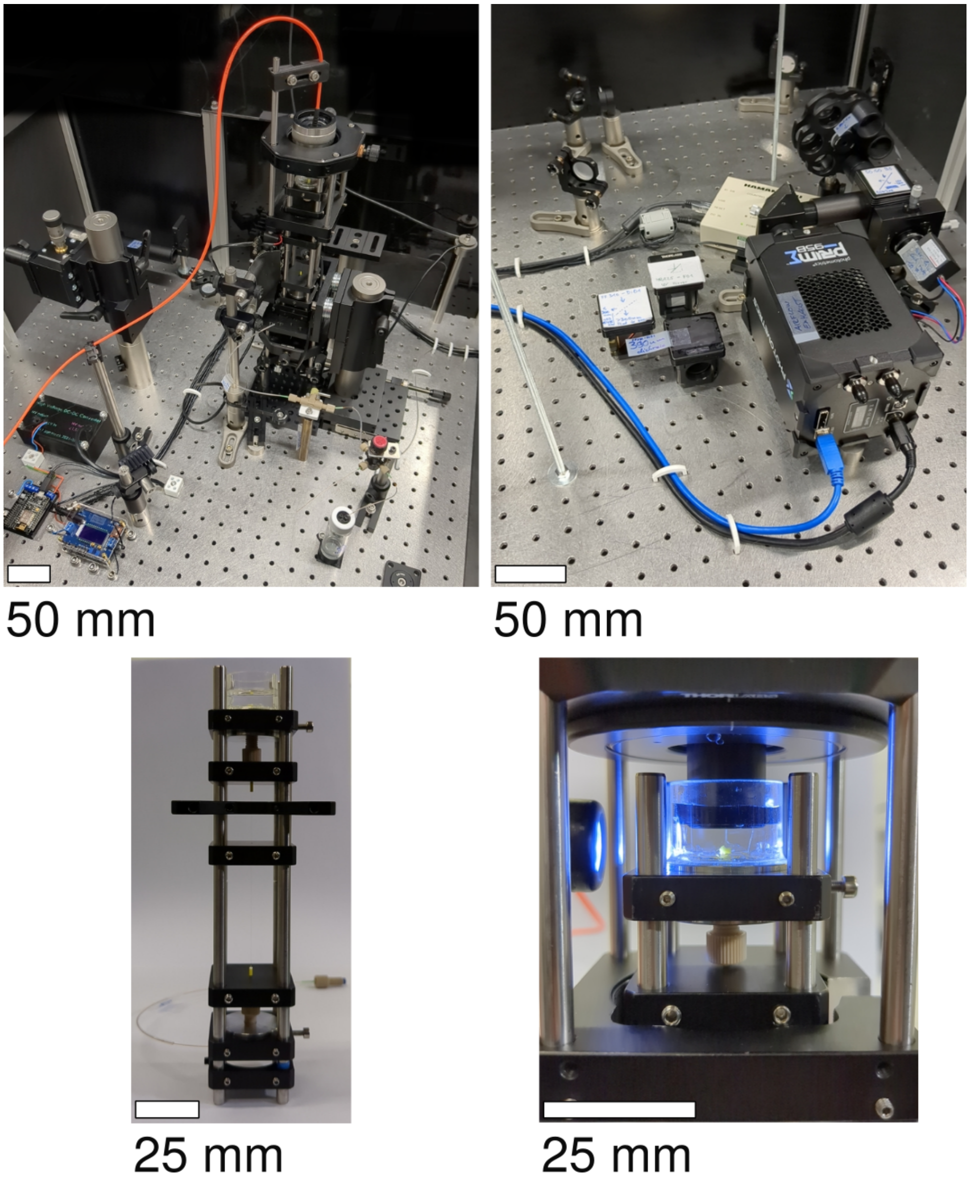

Fig. S1: Setup photos

Top left: Entire setup, built as a vertical tower similar to an inverted microscope. The orange-sleeved multimode fibre delivers the 365 nm light from an LED (butt coupled) to excite a mode in the optofluidic waveguide.

Fig. S2

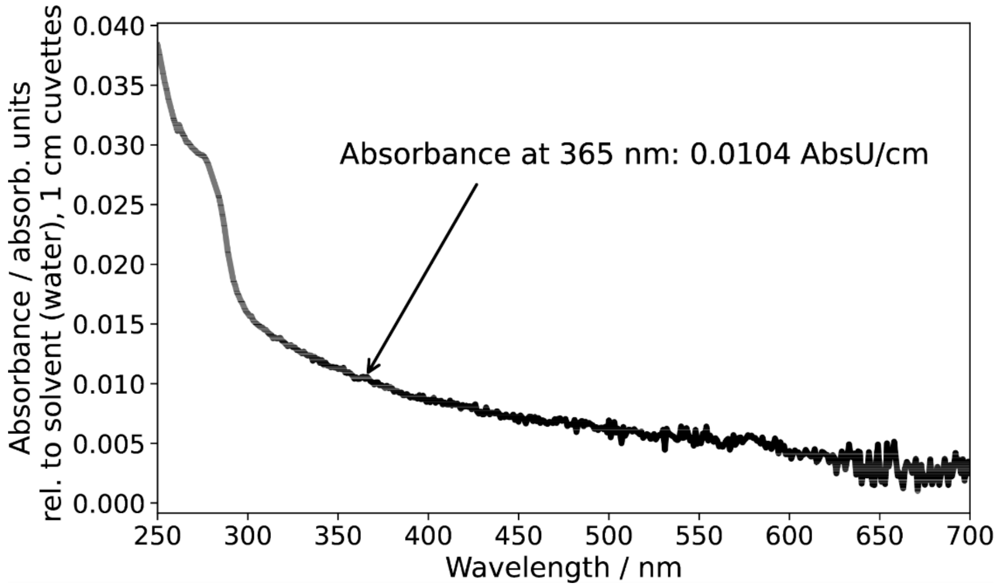

Fig. S2: UV-VIS of the silk aggregates (highest concentration used)

Measured in a large-volume (1.5 mL, 1 cm cuvette) UV-VIS (see Methods).

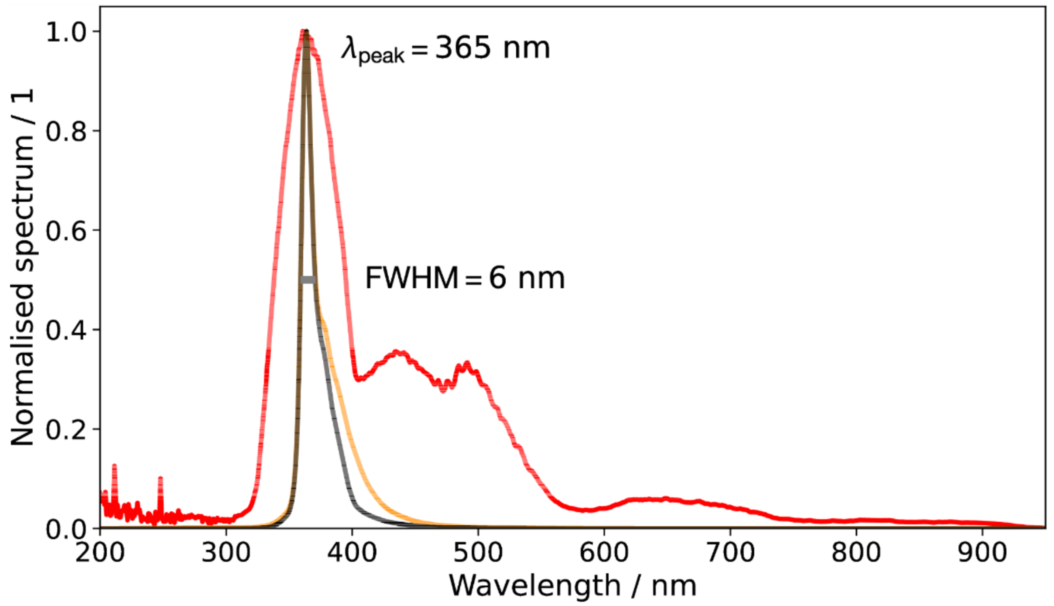

Fig. S3

Fig. S3: Suitability of optofluidic waveguide for the wavelengths used, showing a normalised transmission spectrum for the optofluidic waveguide (**red**), and the spectrum of the 365 nm LED light source used (**orange**). Their product (**black**) is the effective wavelength spectrum used in experiments

The source for the transmission data, and a more detailed and discussion and evaluation of the transmission properties of the optofluidic waveguide, are found in previously published work.[1,2]

Fig. S4

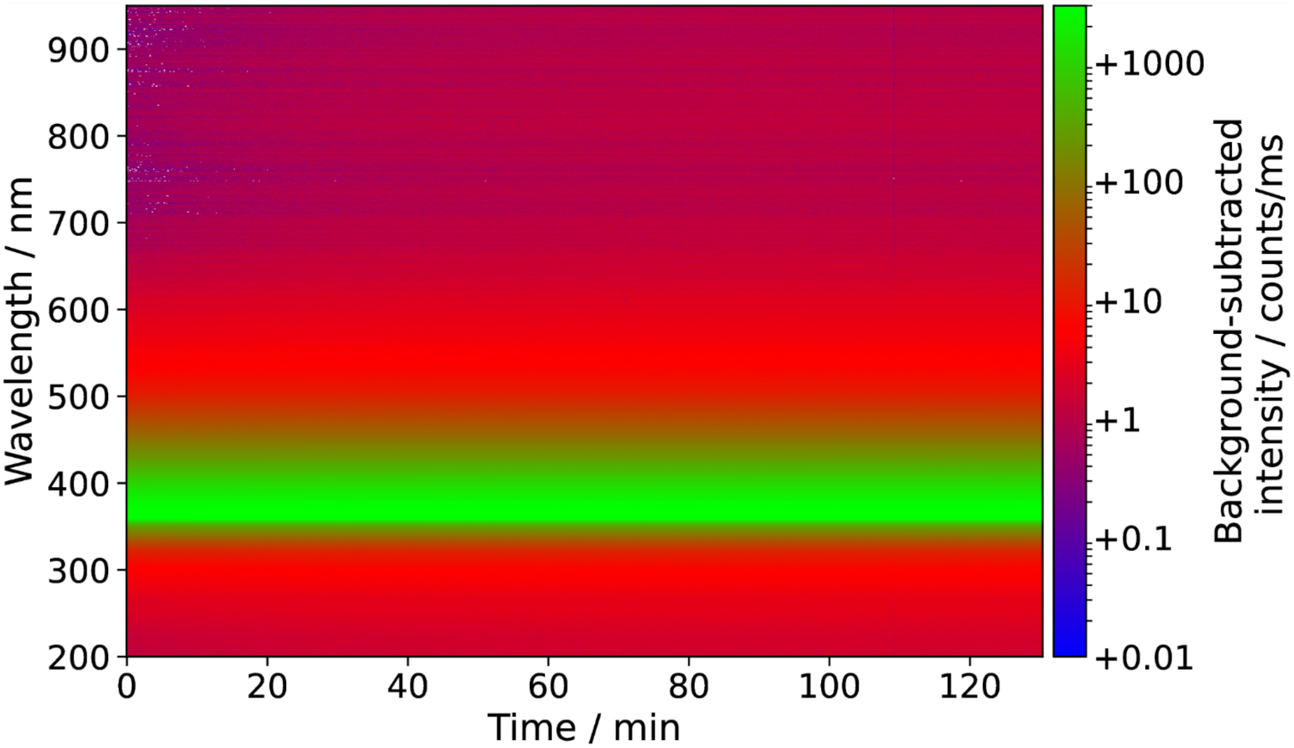

Fig. S4: Spectrum of the 365 nm light source used, as measured at the optofluidic waveguide incoupling plane

The LED was allowed to reach equilibrium temperature before each experiment (> 1h of run time). Under this condition, stability is maintained to within measurement uncertainty for over two hours.

Fig. S5

Fig. S5: Scanning electron micrographs of silk fibroin aggregates

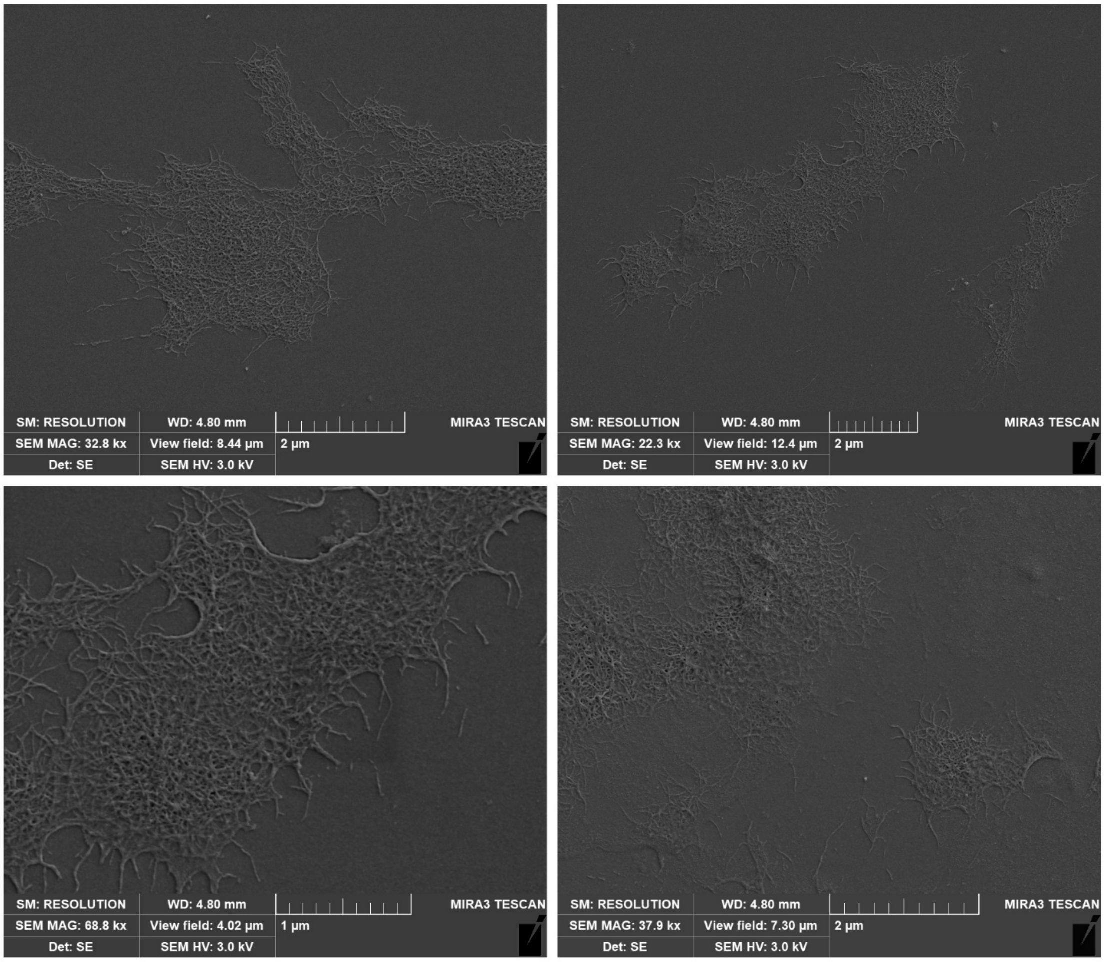

Samples were sputter-coated with platinum to achieve a thickness of 10 nm, following deposition of the sample on a silicon wafer. Electron micrographs were taken on one sample at different positions and magnifications (see image scalebars), acquired with a TESCAN MIRA 3 SEM using 3kV. Images were taken using SE mode.

Fig. S6

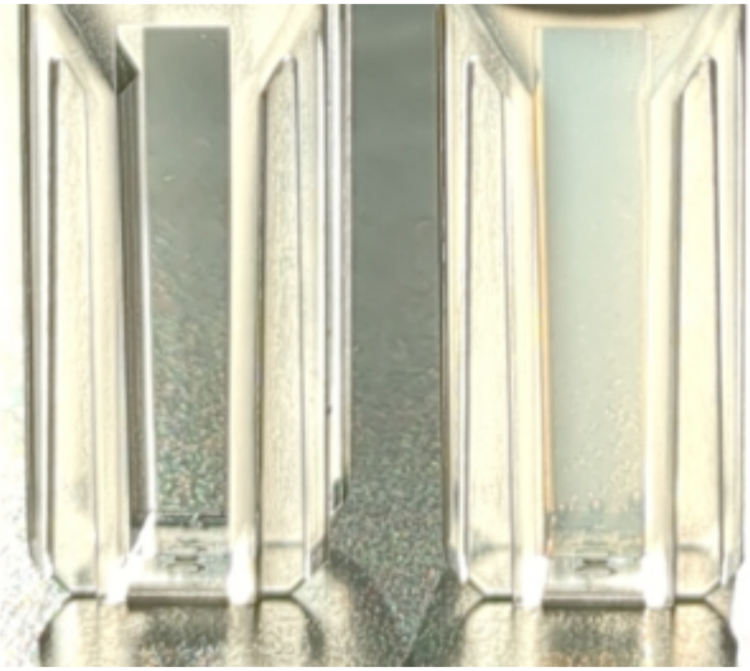

Fig. S6: Photographs of silk solution before (left) and after (right) aggregation, in front of a neutral grey background. Size scale: cuvettes are 1 cm side length

Aggregation was induced with 20% ethanol in 5 g/L silk fibroin solution. The aggregated sample shown here was photographed after no more visible changes to aggregation occur (> 1 week). 1 cm pathlength, 1.5 mL plastic (UV-grade) cuvettes.

Movie S7

Timelapse videos of the in situ aggregation of silk in the HC-PCF

The videos were recorded with frames being 30 s apart in real time (the measurement alternated between taking one absorbance exposure and one fluorescence exposure every 15 s, such that two exposures of the same measurement are 30 s apart). The exposure time was 10 s.

At the chosen playback framerate (10 fps), one second of the video corresponds to 10 frames * 30 s = 5 min in real time.

The recorded images are 16 bit, and when viewed as-is (especially for the ~10x weaker fluorescence images) appear dim and of low brightness. Hence, the following intensity scaling has been performed for the above movies and the images in Fig. 5 which were extracted from it (the timecourse in Fig. 5 was calculated from the unaltered raw images).

Fluorescence
Scale input values from 0.1% and 1% full scale to correspond to the full scale of the output
`ffmpeg filter parameter: -vf "curves=all='.001/0 .01/1'”`

Transmission
Scale input values from 0.1% and 20% full scale to correspond to the full scale of the output
`ffmpeg filter parameter: -vf "curves=all='.001/0 .2/1'”`

Fig. S8

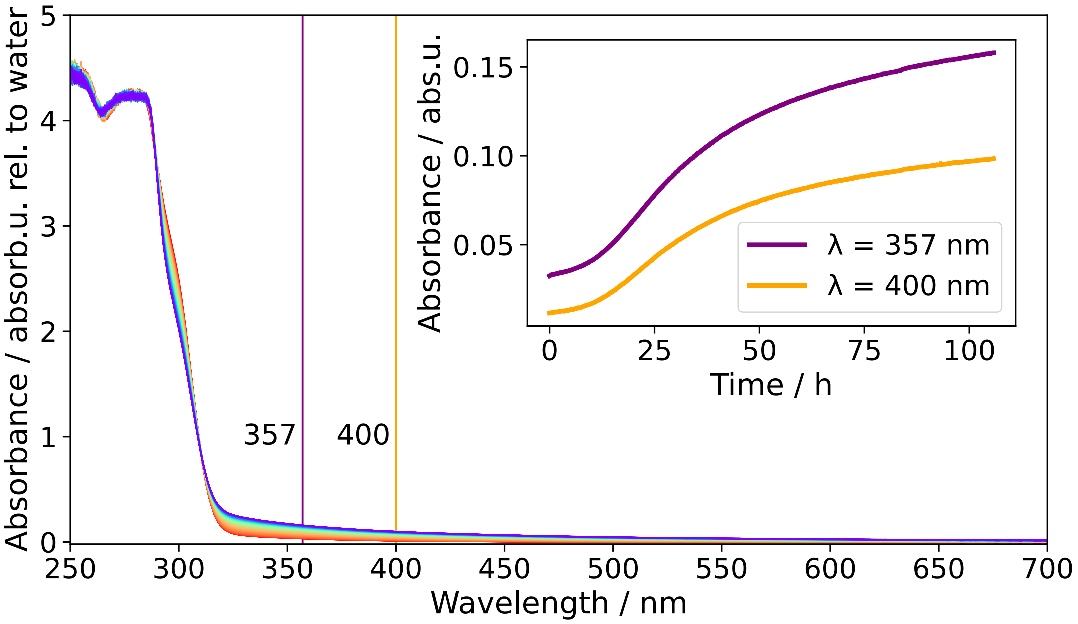

Fig. S7: Silk protein aggregation dynamics in cuvette. Full spectral range graph of the data in Figure 4(a). At wavelengths below 290 nm, the response is limited by the noise floor of the UV-Vis spectrometer. At 300 nm, the absorption is observed to slightly decrease with time, suggesting a decrease in the 275 nm (tryptophan) peak.

References

(1) Heck, J. R.; Miele, E.; Mouthaan, R.; Frosz, M. H.; Knowles, T. P. J.; Euser, T. G. Label-Free Monitoring of Proteins in Optofluidic Hollow-Core Photonic Crystal Fibres. *Methods Appl. Fluoresc.* **2022**, *10* (4), 045008. https://doi.org/10.1088/2050-6120/ac9113.

(2) Heck, J. R.; Miele, E.; Mouthaan, R.; Frosz, M.; Knowles, T.; Euser, T. Label-Free Detection of Proteins with Optofluidic Hollow-Core Photonic Crystal Fibre Sensors. In *SPIE Future Sensing Technologies 2023*; Matoba, O., Valenta, C. R., Shaw, J. A., Eds.; SPIE: Yokohama, Japan, 2023; p 12. https://doi.org/10.1117/12.2643905.
